# Profile of Congenital Hypothyroidism patients at Hasan Sadikin General Hospital, Bandung, Indonesia 2010-2012

**DOI:** 10.1186/1687-9856-2013-S1-P141

**Published:** 2013-10-03

**Authors:** Faisal Bukkar, RM Ryadi Fadil

**Affiliations:** 1 Department of Child Health, Faculty of Medicine, Universitas Padjadjaran, Dr.Hasan Sadikin General Hospital, Bandung, Indonesia

## Background

Congenital hypothyroidism (CH), is caused by inadequate production of thyroid, represent one of the most common preventable causes of mental retardation. Undetected near birth, CH clinically manifests as mental retardation, coarse facial features, poor growth, deafness and neurological abnormalities.

## Objective

To describe characteristics of patient with congenital hypothyroidism diagnosed in Pediatric Department of Hasan Sadikin General Hospital, Bandung, Indonesia

## Methods

We reviewed 26 children with congenital hypothyroidism confirmed by thyroid scintigraphy from October 2010 to June 2012 who came to Dr.Hasan Sadikin General Hospital Bandung, Indonesia.

## Results

Twenty six subjects were diagnosed with congenital hypothyroidism, consisted of 15 (57.7%) girls and 11 (42.3%) boys, mean age 11.42 ±10.35 month. The youngest age when the diagnosis (CH) was established was 2 months and the oldest was 46 months. Thirteen subjects (50%) were referred by primary care pediatrician, 5 subjects (19.2%) by general practitioners, 4 subjects by neuropediatrician and 4 subjects by growth and development clinic. The main presenting complaints in CH were global delayed development (69,2%), constipation (50%), prolonged icteric (15.4%) and growth retardation (13.5%). The most common of clinical appearance were hypotonia (69.2%), coarse faces (46.2%), mottled (34.6%), large fontanel (34.6%), umbilical hernia (23.1%) and macroglossia (26.9%). We found 25 subjects were diagnosed as primary CH and only 1 case with secondary CH. The most common etiology of CH was thyroid agenesis (53.8%), thyroid ectopic (19.2%), thyroid hypoplasia (11.5%) and dyshormonogenesis (11.5%). Decreased fT4 value were found in all subjects (mean 0.553±0.35 ng/dl) and mean TSHs value at presentation was 31.02±20.71 mIU/L. Of the 26 late diagnosed CH cases, 46.% had mental and motor development delay, 23.1% short stature and mental retardation, and 15.4% mental retardation and neurological sequel as complications.

## Conclusion

Late diagnosis of congenital hypothyroidism in children result varied clinical manifestation and had mental retardation, gross motor delay, short stature and neurological abnormalities as complications.

